# IBM Watson Analytics: Automating Visualization, Descriptive, and Predictive Statistics

**DOI:** 10.2196/publichealth.5810

**Published:** 2016-10-11

**Authors:** Robert Eugene Hoyt, Dallas Snider, Carla Thompson, Sarita Mantravadi

**Affiliations:** ^1^ College of Health Department of Health Sciences and Administration University of West Florida Pensacola, FL United States; ^2^ College of Science and Engineering Department of Computer Science University of West Florida Pensacola, FL United States; ^3^ College of Education and Professional Studies Community Outreach Research and Learning (CORAL) Center University of West Florida Pensacola, FL United States

**Keywords:** data analysis, data mining, machine learning, statistical data analysis, natural language processing

## Abstract

**Background:**

We live in an era of explosive data generation that will continue to grow and involve all industries. One of the results of this explosion is the need for newer and more efficient data analytics procedures. Traditionally, data analytics required a substantial background in statistics and computer science. In 2015, International Business Machines Corporation (IBM) released the IBM Watson Analytics (IBMWA) software that delivered advanced statistical procedures based on the Statistical Package for the Social Sciences (SPSS). The latest entry of Watson Analytics into the field of analytical software products provides users with enhanced functions that are not available in many existing programs. For example, Watson Analytics automatically analyzes datasets, examines data quality, and determines the optimal statistical approach. Users can request exploratory, predictive, and visual analytics. Using natural language processing (NLP), users are able to submit additional questions for analyses in a quick response format. This analytical package is available free to academic institutions (faculty and students) that plan to use the tools for noncommercial purposes.

**Objective:**

To report the features of IBMWA and discuss how this software subjectively and objectively compares to other data mining programs.

**Methods:**

The salient features of the IBMWA program were examined and compared with other common analytical platforms, using validated health datasets.

**Results:**

Using a validated dataset, IBMWA delivered similar predictions compared with several commercial and open source data mining software applications. The visual analytics generated by IBMWA were similar to results from programs such as Microsoft Excel and Tableau Software. In addition, assistance with data preprocessing and data exploration was an inherent component of the IBMWA application. Sensitivity and specificity were not included in the IBMWA predictive analytics results, nor were odds ratios, confidence intervals, or a confusion matrix.

**Conclusions:**

IBMWA is a new alternative for data analytics software that automates descriptive, predictive, and visual analytics. This program is very user-friendly but requires data preprocessing, statistical conceptual understanding, and domain expertise.

## Introduction

Studies have shown that physicians tend to lack data analytical expertise, most likely due to insufficient training in statistics while in medical school or not using statistics on a regular basis [1,2]. Gigerenzer et al conclude that there is “collective statistical illiteracy” in health care today [3]. Moreover, there is evidence that teaching statistics to students outside of their health care specialty area has been relatively unsuccessful [4]. Contrastingly, the need for data analytical expertise by health care personnel has grown exponentially, given the explosion of health care data in the past decade [5]. The situation is complicated by a lack of trained data scientists to analyze new data. According to McKinsey Global Institute, there will be a shortage of 140,000 to 180,000 data scientists by 2018 [6].

Therefore, new tools are needed to assist health care workers in analyzing health data. Clinicians and other health care workers would benefit from tools that could produce descriptive, predictive, and visual analytics more rapidly and easily than tools currently available in most analytical software packages and with little training required for users.

One new tool with potential benefit to health care workers is IBM Watson Analytics (IBMWA), introduced in 2015. Unlike the Watson that won *Jeopardy* in 2011, this platform is not based on cognitive computing; instead, it is based on a practitioner approach to using advanced statistics [7]. IBMWA is available in free, personal, and professional versions. An IBMWA Academic Program was also released in 2015 that provides free access to the professional version of the program for faculty and students, if used for noncommercial purposes [8].

In this paper, we will report on the features of IBMWA and discuss how this software subjectively and objectively compares with other data mining programs.

## Methods

### Software Packages

#### IBM Watson Analytics

IBMWA utilizes advanced statistics based on Statistical Package for the Social Sciences (IBM SPSS, IBM Corporation), and the statistical tests used are enumerated in Table 1 [7,9]. The available tests were chosen to provide analysis of nonparametric (nominal and ordinal) and parametric (interval and ratio) types of data. Statistical significance is presented with standard *P* values in addition to effect size.

The features available in the professional (and academic) versions are summarized in Table 2. IBMWA also provides access to additional data sources inherent in social media (such as Twitter feeds) and an enhanced ability to share datasets.

New features are added frequently. IBMWA has 4 basic sections: refine, explore, predict, and assemble that are described in the following paragraphs [7].

##### Refine

The refine section is used for data exploration and manipulation. This is a logical starting point to examine any dataset. Here, spreadsheet rows and columns are displayed. Attributes can be renamed, calculations can be embedded, and data can be placed in groups or hierarchies for subgroup analysis. Attributes can be organized into ascending or descending order, and a data score and percent missing data per column is displayed.

##### Explore

The explore section is used for descriptive analytics and is demonstrated in the next section. Using the natural language processing (NLP) function of Watson Analytics, a user can enter other questions in the search window. In addition to the map view, data can be represented in tree, heat, grid, area, bar, bubble, line, pie, and categorical charts. IBMWA recommends the optimal display type. The page can be saved for a dashboard, or shared via email, social media, or downloaded. In addition, a hyperlink can be created for the page for remote viewing.

##### Predict

The predict section is used for predictive analytics. The user selects the target attribute and IBMWA generates a predictive strength. For categorical targets, the predictive strength is the proportion of correct classifications, and for continuous targets it is 1-relative error. Data quality is rated at the top of the page with mention of any outliers, skewed distributions, and missing data. A user can request an analysis with a single factor, 2 factors, or all factors. The predict results can be saved and shared, similar to the explore function. Hyperlinked statistical details are available that provide the statistical test used, the statistical significance, and effect size.

##### Assemble

The assemble section allows for data visualization and dashboard creation. This function creates dashboards, infographics and slide shows by simply dragging and dropping data into the active panes. Multiple choices exist for users to have options for representing or displaying data [7].

#### IBM Statistical Package for the Social Sciences

SPSS is a comprehensive statistical package available in standard, professional, and premium versions. Table 3 outlines the features of the professional version [9]. The basic or standard SPSS software package provides a user-friendly, menu-driven approach for analyzing data without the need for programming skills or formula-driven guidelines, options for applying multiple analyses, and generating graphs and visuals to meet the needs of social science researchers with minimal statistical preparation skills. Unlike Microsoft Excel, SPSS provides a direct use and understanding of variables and analyses relative to research principles and interpretation options rather than a formula-driven and spreadsheet (location-dependent) approach to data analyses.

**Table 1:** IBM Watson statistical tests.

Statistical test	Indication
Analysis of variance (ANOVA)	ANOVA tests mean differences among 2 or more groups and whether the mean target value varies across combinations of categories of 2 inputs; If the variation is significant, there is an interaction effect
Asymmetry index	Ratio of skewness to the standard error
Chi-square automatic interaction detector classification tree	Decision tree using chi-square for prediction
Chi-square automatic interaction detector regression tree	Decision tree using chi-square and regression for prediction
Chi-square tests	Using chi-square to compare frequencies in groups, independence, and marginal distributions
D’Agostino’s K-squared test of normality	Determines if normal distribution is present
Distribution test	Chi-square test compares conditional distributions with overall distribution
Fisher r-to-t test	Transforms Pearson’s *r* to *t* test for significance
High low analysis	Partitions categories into high or low groups for analysis
Influence test	Chi-square test determines whether the number of records in a group is significantly different from the expected frequency.
Model comparison test	Tests whether the key driver has an effect on the logistic regression
Paired samples *t* test	Dependent *t* test checks whether the means of 2 continuous fields are statistically different or if there is a change in means over time for one group
Unusually high or low analysis	Determines which categories or combinations of categories across categorical fields have unusually high or low target mean values

**Table 2:** Features of IBM Watson Analytics Professional.

Features	IBM Watson Analytics Professional
Maximum number of rows per dataset	10,000,000
Maximum number of columns per dataset	500
Input in .csv, .xls or, .xlsx formats	Uploaded from PC, Dropbox, IBM Cognos, Box, and Microsoft OneDrive
Data connections	IBM Cognos BI server, IBM dash DB, IBM DB2, IBM SQL, Microsoft SQL server, MySQL, Oracle, and PostgreSQL
Storage	100 GB; can be increased in increments of 50 GB

**Table 3:** Features of SPSS.

Tool	Function
Core stats and graphics	Standard statistical tests for nominal, ordinal, interval, and ratio data
Integration with R and Python languages	Expands programmability involving additional languages
Multiple linear and mixed modeling	Analyze complex relationships
Nonlinear regression	Predictions on nonlinear data
Simulation modeling	Build risk models when inputs are uncertain
Geospatial analytics	Integrate and analyze time and location data
Customized tables	Analyze and report on numerical and categorical data
Charts, graphs, and mapping	Assist reporting capabilities
Missing value analysis	Address missing data, imputation, etc
Advanced data preparation	Identify data anomalies
Decision trees	Identify group relationships to predict future events
Forecasting techniques	Predict trends with time-series data
SPSS Text Analytics	This add-on complementary software package accompanies SPSS to provide qualitative data analyses and visuals for quantitative data simultaneously analyzed with SPSS

**Table 4:** Features of Microsoft Excel Analysis ToolPak.

Tool	Function
Analysis of variance (ANOVA)	Determines variance on single or multiple factors and mean differences among 2 or more groups
Correlation	Determines if a pair of variables are related
Covariance	Determines if a pair of variables move together and mean differences in 2 or more groups when controlling for initial group differences
Descriptive statistics	Determines central tendency and variability in the data
Exponential smoothing	Predicts a value based on prior forecast
*F* test for 2-sample variances	Performs a 2-sample *F* test to compare population variances and mean differences relative to variability testing
Fourier analysis	Transforms time-based patterns into cyclical components
Histogram	Calculates frequencies of values in dataset
Moving average	Forecasts values based on prior averages
Random number generation	Fills a range with independent random numbers
Rank and percentile	Creates a table with ordinal and percentile ranks and used with chi-square analyses
Regression	Linear regression based on “least squares” method
Sampling	Creates a sample from a population
*t* test	Tests for equality of population means, with equal and unequal variances based on 1-group or 2-group datasets
*z* test	Performs a 1-sample *z* test for population comparison or a 2-sample *z* test for means with known variances

**Table 5:** Features of Microsoft SQL Server Analysis Services.

Tool	Function
Multiple data inputs	Use tabular data, spreadsheets, and text files
Data management	Data cleaning; management; and extract, transform, and load
Model testing	Use cross-validation, lift, and scatter charts
Data mining algorithms	Clustering, Naïve Bayes, decision trees, neural networks, regression, and association rules
Scripting language support	Mining objects are programmable

**Table 6:** Features of Waikato Environment for Knowledge Analysis.

Tool	Function
Preprocess	Descriptive statistics and ability to preprocess data; Data from .csv and .arff files, web data, database data, and ability to generate artificial data
Classify	Classify data from Bayes, neural networks, regression, decision trees, production rules, and other algorithms
Cluster	12 clustering algorithms, to include the common simple k-means
Associate	Association rules for pattern recognition in data
Select attributes	Searches for best set of attributes in dataset
Visualize	Visualization of data into graphs, etc

#### Microsoft Excel Analysis ToolPak

The ToolPak is a spreadsheet add-on that provides the features found in Table 4 [10]. The use of Microsoft Excel Analysis ToolPak requires the user to acquire and install the add-on as well as cognitively account for the differences between the ToolPak spreadsheet-driven program (involving placeholders for data locations requiring user-generated formulas to drive analyses) and SPSS or IBMWA analysis-driven programs, whereby data and locations are defined variables with analyses formulas inherent in the software programs.

#### Microsoft SQL Server Analysis Services

Microsoft SQL Server Analysis Services is an integrated platform for data mining that uses relational or cube data in multiple formats to provide predictive analytics. A summary of the features is provided in Table 5. Mining choices include clustering, neural networks, decisions trees, and custom plug-in algorithms. Because Analysis Services is a component in SQL Server’s suite of business intelligence tools, it integrates easily with the SQL Server database engine and reporting services component. Analysis Services provides a confusion matrix, but requires the user to manually calculate classification accuracy measures, such as precision and recall. Additionally, Analysis Services generates a lift chart, in lieu of receiver operating characteristic curves [11].

#### Waikato Environment for Knowledge Analysis

Waikato Environment for Knowledge Analysis (WEKA) is a free machine-learning software platform developed by the University of Waikato in New Zealand. This popular program is used for data mining utilizing primarily classification and clustering tools consisting of rules, decision trees, and multiple other algorithms. WEKA calculates true positive rates, false positive rates, precision, and recall. WEKA will also create the receiver operator characteristic curves and area under the curve [12]. A summary of the features in WEKA is provided in Table 6.

### Data

The datasets used for comparing IBMWA and prevailing software are publicly available datasets.

#### County Health Rankings for Florida

The dataset used to demonstrate IBMWA features was derived from the publicly available 2014 County Health Rankings for the state of Florida [13]. The dataset was altered by merging demographics with health factors and deleting confidence intervals and *z*scores to reduce the number of attributes (columns). This dataset ranks counties in each state by health outcomes and health factors, with health factors categorized as health behavior, clinical care, social and economic factors, and physical environment. The data matrix was comprised of columns consisting of 41 common health factor attributes and demographics; the rows consisted of the 67 Florida counties.

#### Heart Disease Data

The dataset used for comparison among the analytical software packages was derived from a well-known and validated machine-learning repository [14]. The selected dataset focused on the diagnosis of heart disease (presence or absence) based on 13 common cardiac risk factors or test attributes (columns) and 270 instances or rows (patients). The thallium test (cardiac scan) attribute had 3 subcategories: 3 = normal test, 6 = fixed defect, and 7 = reversible defect.

## Results

### Use Cases

#### Explore Option

**Figure 1:**
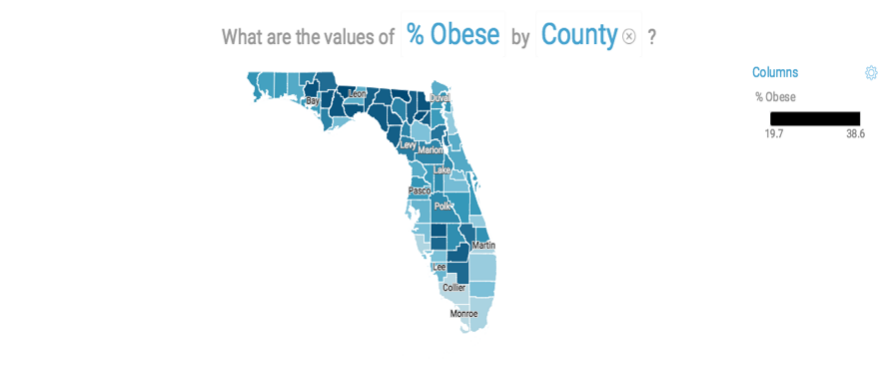
The % obese by Florida county.

The use cases shown in this section are generated using the sample data file named 2014 County Health Rankings for the State of Florida. In the explore section, which is used for descriptive analytics, Watson Analytics automatically generated 10 questions based on the data such as “What is the breakdown of % obese by county?” A map of all Florida counties was automatically generated (without user prompting) with % obese noted for each county (Figure 1), as well as the range. The user can mouse over each county for specific data.

**Figure 2:**
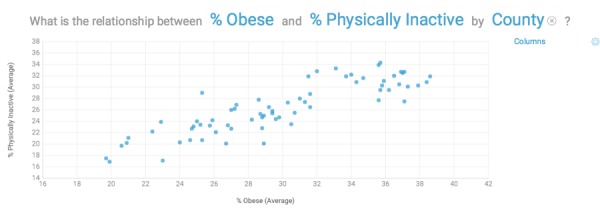
The relationship between % physically inactive and % obese by county.

A user can also enter questions in the search window by leveraging the NLP function of Watson Analytics, for example, “What is the relationship between % physically inactive and % obese by county?” (Figure 2)

**Figure 3:**
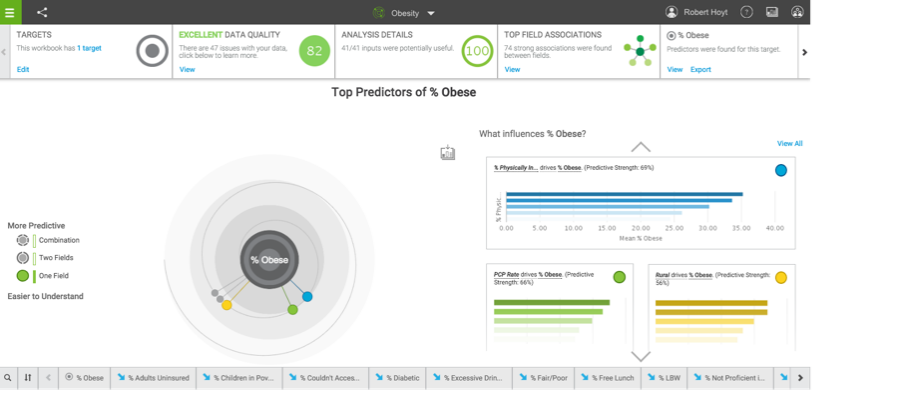
Predictors for factors related to % obese.

**Figure 4:**
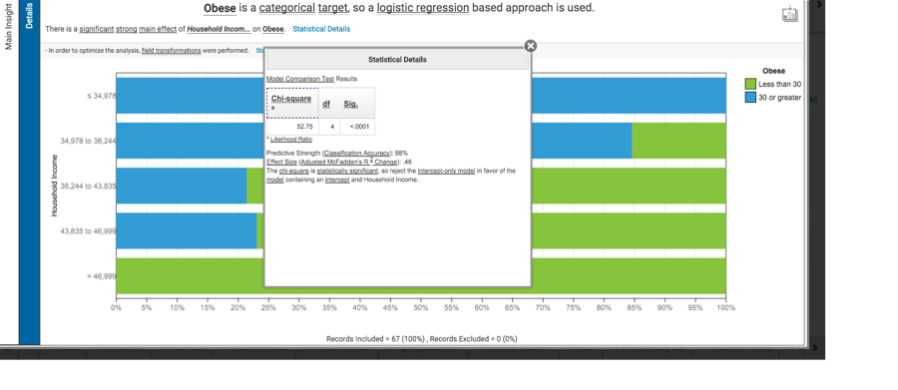
Predict option for nominal category of less than or greater than 30% obese by county.

**Figure 5:**
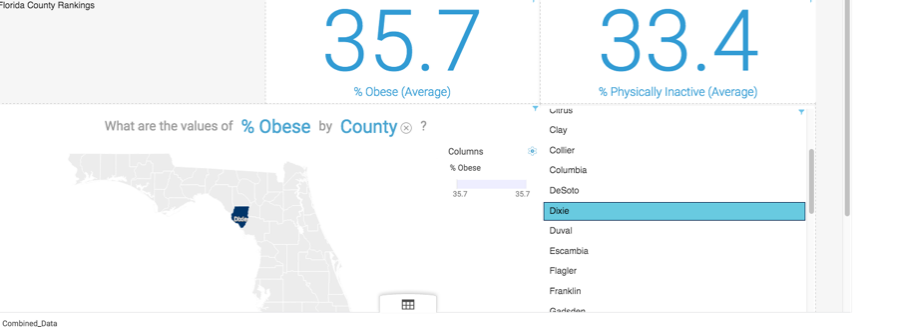
Dashboard of Florida County Health Rankings.

#### Predict Option

The predict option in IBMWA is utilized for predictive analytics. In our analysis of the 2014 County Health Rankings for the state of Florida, 74 associations were noted at the top of the page. The attribute “children in poverty” was associated with “teen birth rate.” Select “statistical details” and a Pearson correlation of .79 with *P*<.001 and effect size of 0.63 was noted.

When % obese was selected as the target, predictions were automatically generated. The top predictor for “% obesity” was “% physically inactive” at 69%, but IBMWA recommended the addition of “% African-American,” which increased the predictive ability to 85%. A screenshot of the predict function results is shown in Figure 3.

The “% obesity” column of attributes was then subdivided into counties with less than or more than 30% obesity reported and the predict function was reexecuted. This second analysis used logistic regression and produced household income as the strongest predictor at 88% predictive strength. A chi-square analysis comparing the categorical variables demonstrated the following: *P*<.001, effect size = .46 (Figure 4).

#### Assemble Option

The assemble option contains functionality to create dashboards, infographics, and slide shows. An example of an IBMWA interactive dashboard display using the dataset, 2014 County Health Rankings for the state of Florida reflecting the “% obesity” by Florida County is depicted in Figure 5. The county dropdown list is active; such that once a county is selected, the percentages change and correspondingly the map changes.

### Comparative Study

The results from the comparative study among the software packages are presented in the following subsections. The same heart disease dataset was used as the input to each software package and each package provides differing statistics and measures which are summarized in Table 7.

#### IBM Watson Analytics

The IBMWA software conducted a logistic regression for classification purposes. When the target attribute of heart disease (present or absent) was used in IBMWA, it revealed that the thallium test had a predictive strength of 76% (percent correct classification). The thallium test attribute had 3 subcategories: 3 = normal, 6 = fixed defect, and 7 = reversible defect. Based on either normal exam or reversible defect on thallium testing, the chi-square test revealed *P*<.001 and an effect size of 0.48 for the normal test and 0.63 for the reversible defect.

When a full model (3 variables) analysis is conducted with logistic regression, the software also conducts a likelihood ratio test (chi-square) to determine if the addition of the variables improves the fit of the model. Predictive strength increases to 80% (percent correct classification) and statistical significance of the target predictor of thallium reduced. Interactions between thallium and the number of vessels calcified on fluoroscopy were not significant, *P*<.09. The likelihood ratio test (χ^2^_1_= 11.08, *P*<.08) was not statistically significant to the 5% significance level, and thus the reduced model of thallium alone and heart disease is the best fit.

#### IBM Statistical Package for the Social Sciences

Binary logistic regression with heart disease as the dependent variable and thallium as a single predictor was conducted. As confirmed in the IBMWA results, predictive strength and percent correctly classified increases as more variables are included in the regression; however, statistical significance reduces.

Logistic regression (LR) with 3 predictors—thallium, number of vessels calcified on fluoroscopy, and the interaction effect—was conducted, illustrating that the predictive strength of the model was 78%, and the interaction effect was not significant. The number of vessels calcified by fluoroscopy and the thallium test variables were statistically significant with *P*=.04 and *P*<.001, respectively. The LR test compared with the intercept only model was significant, with χ^2^_3_=120.5 and *P*<.001, indicating that the 3 variable model improved model fit over the intercept only model.

Thereafter, forward selection using the LR test was also conducted for appropriate variable selection, reducing collinearity and demonstrating model fit. By the end of the stepwise forward regression concerning all variables, the LR test indicated that thallium remained a statistically significant predictor, as well as gender, type of chest pain, electrocardiogram results, exercise-related angina, ST wave depression, and number of vessels calcified by fluoroscopy. Percent correctly classified increased to 90%. The variables gender (χ^2^_1_=3.9, *P*=.049), exercise-related angina (χ^2^_1_=5.7, *P*=.02), and electrocardiogram results were statistically significant at the 5% level, whereas, types of chest pain (χ^2^_1_=13.3, *P*<.001), ST wave depression (χ^2^_1_=11.7, *P*=.001), number of vessels calcified by fluoroscopy (χ^2^_1_=19.9, *P*<.001), and thallium (χ^2^_1_=15.1, *P*<.001) were statistically significant at the 1% level. Comparing this full model with the intercept only model, it was found that χ^2^_1_= 77.8 and *P*<.001. These results illustrate that the full model had a better model fit than both the intercept only model. However, IBMWA uses the LR test to compare models with the reduced fit single predictor model as the default setting, whereas SPSS uses the baseline, intercept only model as the default setting for LR test comparison.

A chi-square analysis was also performed using SPSS with a resulting likelihood ratio of 78% for comparison purposes. Based on the normal exam or reversible defect on thallium testing, the chi-square test revealed a significant relationship (χ^2^_1_= 76.1, *P*<.001) and an effect size of 0.53 for the normal test and 0.67 for the reversible defect test.

#### Microsoft Excel Analysis ToolPak

The ToolPak software can only conduct linear regression, not logistic regression for classification.

Analysis was not performed because a chi-square test would have to be manually run between the target attribute and each column. The expected values would need to be calculated and run against the actual values to arrive at the chi-square result and *P* value. This is very labor intensive compared with the other platforms tested.

#### Microsoft SQL Server Analysis Services

Data were analyzed using a decision tree and neural network to compare for classification accuracy. To train the classifier models, 70% of the data was used, whereas the remaining 30% was held out for testing. The decision tree algorithm was chosen because of the ease of understanding the results, while a neural network was selected because of the ability to generally produce better classification results. The decision tree yielded a sensitivity of 0.80 and specificity of 0.78, while neural networks yielded a sensitivity of 0.77 and a specificity of 0.92. Both algorithms have parameters that can be adjusted to improve classification accuracy; however, these parameters need to be adjusted cautiously to avoid “overfitting” the model.

#### Waikato Environment for Knowledge Analysis

A J48 decision tree was used as the algorithm with 10-fold cross validation. The outcome was correctly classified 78% of the time. The precision for the presence of heart disease was 0.931 and recall (sensitivity) was 0.628. Precision for the absence of heart disease was 0.692 and the recall was 0.947.

### Summary

These preliminary informal analyses indicate that the 4 analytical programs provide similar results using the same dataset. WEKA does provide a confusion matrix, Kappa statistic, and receiver operator characteristics curve area statistic, with neither of these analytics supplied by IBMWA. WEKA, in contrast to IBMWA, includes more than 50 different algorithms, without any recommendations regarding the optimal choice.

**Table 7:** Results of the comparison of different analytical packages.

Software package	Results
IBM Watson Analytics	Using logistic regression, the thallium test had a predictive strength of 76% (percent correct classification); chi-square test revealed *P*<.001 and an effect size of 0.48 (normal test) and 0.63 (reversible defect)
Statistical Package for the Social Sciences	Using logistic regression, a full model with thallium, number of vessels calcified on fluoroscopy, and interaction test increased the predictive strength to 78%; however, a statistically insignificant chi-square test proved that the single model using thallium had the better model fit. χ^2^_1_= 76.1, *P*<.001; effect size 0.53 (normal test) and 0.67 (reversible defect)
SQL Server Analysis Services	Decision tree analysis yielded a sensitivity of 0.80 and specificity of 0.78, while neural networks yielded a sensitivity of 0.77 and a specificity of 0.92
Waikato Environment for Knowledge Analysis	Decision tree precision for presence of heart disease was 0.93 and recall (sensitivity) was 0.63; precision for absence of heart disease was 0.69 and recall was 0.95

## Discussion

### Principal Findings

According to Dr. Bill Hersh, “Analytics and related activities are the future of clinical informatics, realizing the goal of my definition of the field, which is the use of information to improve individual health, health care, public health, and biomedical research [15].” To achieve this goal, we will need a well-trained workforce and supportive analytical tools.

IBMWA is an analytical program based on SPSS that automatically generates descriptive, predictive, and visual analytics. This approach is compatible with “greater statistics” proposed by John Chambers in 1993 [16]. Traditional statistics can be laborious when manual computation of complicated formulas is required. Steps to simplify this approach will likely be well received. One could argue that automating analytics is a logical progression, similar to using electronic medical calculators, instead of paper calculations or using the “what you see is what you get” program to create webpages, rather than requiring html programming. This program will likely open the analytics playing field to a larger audience of health care workers.

The learning curve for IBMWA is much less steep than Microsoft Excel Analysis ToolPak, SPSS, WEKA, or Microsoft Server Analysis Services. Instead of needing an extensive background in statistics to decide on the statistical method of choice, this is performed automatically for the user. A busy health care worker might use this program to gain preliminary results and then consult an expert in data science or statistics.

**Figure 6:**
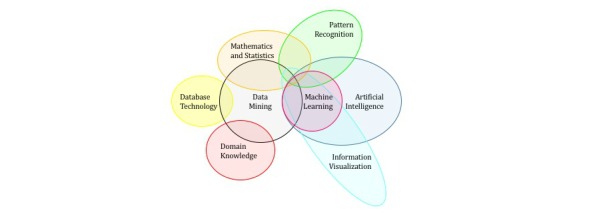
Overlap between statistics and machine learning.

IBMWA is able to handle very large datasets and applies the most common statistical tests required, but does not perform data mining using machine-learning techniques, such as neural or Bayes networks, and is not appropriate for many big data sets. The statistical approach is, however, complimentary to the machine-learning approach. Statistical modeling using a program such as IBMWA usually involves smaller datasets, a hypothesis, and a list of assumptions. Machine-learning, on the other hand, can handle larger datasets, and does not require the same hypotheses or assumptions. An overview of the existing software programs supports IBMWA as belonging in the overlap region between data mining and statistics as demonstrated in Figure 6. In addition, this preview of the use of multiple software programs initiates a scholarly conversation concerning the considerable overlap between statistics and machine learning as exemplified in Figure 6 [17].

A comparison of IBMWA with 3 other data analytical software resulted in similar, but not identical results. We did not report results with Microsoft Excel due its inability to perform logistic regression and the labor intensive nature of the analysis.

IBMWA may be a helpful adjunct approach to teaching both statistics and data mining, given its speed, functionality, and ease of use. Case studies could be presented in the domain of interest and both the clinical results and statistical methods could be discussed. The average user would be able to see missing data, skewed distributions, and outliers with minimal effort. Large datasets are amenable to data analyses and quick response outcomes using IBMWA- a key element in teaching and learning statistical concepts. In the University of West Florida, IBMWA is used to augment understanding and applications of statistical concepts in several health informatics and computer science courses. The use of NLP to help explore, analyze, and visualize datasets would be helpful for most graduate students, regardless of field.

### Limitations

For classification purposes with categorical data, IBMWA does not offer the user a choice of statistics, but rather selects the approach for the user (eg, logistic regression). IBMWA did not aid users in interpreting results, especially the impact of unrelated variables and highly correlated variables (multicollinearity). The aim of IBMWA is ease of use but correct interpretations might be eluded due to the lack of odds ratios, confidence intervals, and an explanation for seemingly counterintuitive results.

The combination option in IBMWA makes it easier for users to create complex models, evaluating the effect of up to 3 variables on the dependent variable. Users may not be able to determine the best model; for the average user, it becomes harder to determine if the right model is chosen—with the appropriate theoretically related variables. For example, with multiple predictors in a classification analysis, the chi-square value provided indicates if model fit improves with complexity and increased variables (likelihood ratio test result). Predictive strength increases as additional variables are added; however, this is counterintuitive and easily confused as decreased statistical significance (low chi-square value) with increased predictors—leading to inaccurate interpretation. In IBMWA, as variables are added and complexity increases, the model starts to become confusing to interpret without taking the extra step of checking the fine print definitions; the chi-square and *P-* value provided in a full model is actually for comparing goodness of fit of a statistical model (LR test), not a measure of variable statistical significance. Users may initially focus on the fact that predictive strength will increase each time another variable is added to the model, resulting in a model that is overly complex with unrelated variables. For early health data analysts, the issue of highly correlated variables (multicollinearity) and model fit is hard to detect and interpret. Too many variables can result in predicting noise, rather than the dependent variable (overfitting). The LR test acts as a warning sign for users—don’t add more variables in the model just to increase predictive strength.

In addition, IBMWA provides for ease of data exploration, creating a conundrum for the average user—with such ease of data mining and exploration, eventually the user will detect chance correlations between variables that appear to be significant relationships. IBMWA allows the average user to depend on statistical significance as a measure of the strength of the correlation, as well as the correct model; however, a correctly specified model might have insignificant, theoretically relevant predictors. The IBMWA software does not provide statistical significance for each predictor but only the results of the model comparison test (likelihood ratio test).

Limitations exist with every analytical platform. Data preprocessing (imputation and data quality) is needed and is critical for success. It is estimated that about 80% of the time spent analyzing data, is spent exploring and preparing the data for analysis [18]. Specifically, data may need cleaning, integration, reduction, or transformation before analysis procedures [19]. In addition, data must be of sufficient quality and volume to answer a question. Finally, domain expertise and appropriately prepared researchers are needed to ask appropriate questions and interpret results, regardless of the analytical platform used.

### Conclusions

IBMWA is a new and interesting analytical tool that may be of value to multiple types of health care workers; however, no statistical program will replace the time needed for preprocessing data and asking pertinent questions regarding the dataset, but the time spent on analytical processes will be greatly expedited. The IBMWA approach needs to be compared and contrasted with other approaches and by a diverse group of users to better understand its role within the analytics realm. Clearly, IBMWA has limitations but IBM is making frequent changes to this program so users can expect more functionality in the future. Additionally, IBMWA may motivate educators and practitioners to question if it is potentially effective as an adjunct in teaching statistics and analytics in health care.

## Abbreviations

ANOVA: Analysis of variance
IBM: International Business Machines Corporation
IBMWA: IBM Watson Analytics
LR: Logistic regression
NLP: natural language processing
SPSS: Statistical Package for the Social Sciences
WEKA: Waikato Environment for Knowledge Analysis
